# Nontoxic, Eco-Friendly, Low-Cost Spinning of Submicrometric Fibers from Water-Soluble Polymers Using Protic Ionic Liquids

**DOI:** 10.1021/acsomega.5c13582

**Published:** 2026-08-06

**Authors:** Vinícius D. Silva, Fabio E. F. Silva, Elizeth O. Alves, Rebecca S. Andrade, Maurício C. Menezes, José E. Soares Filho, Miguel Iglesias, Daniel A. Macedo, Eliton S. Medeiros, Thiago A. Simões

**Affiliations:** † Department of Fundamental Chemistry, Institute of Chemistry, University of São Paulo, São Paulo, São Paulo 05508-000, Brazil; ‡ Materials Science and Engineering Postgraduate Program, 28097 UFPB, João Pessoa 58051-900, Brazil; § Engineering Campus − UACSA, Federal Rural University of Pernambuco (UFRPE), Cabo de Santo Agostinho, Pernambuco 54518430, Brazil; ∥ CETENS, 186074 Federal University of Recôncavo da Bahia, Feira de Santana 44042-280, Brazil; ⊥ Department of Chemical Engineering, Federal University of Bahia, UFBA, Salvador 40210-630, Brazil; # Laboratory of Materials and Biosystems (LAMAB), UFPB, João Pessoa 58051-900, Brazil

## Abstract

Ionic liquids (ILs) are widely utilized in various scientific fields and technologies, offering environmentally friendly alternatives to toxic volatile solvents. In particular, protic ionic liquids (PILs), a subset of ILs, boast advantages such as simple synthesis, low cost, and biodegradability. ILs are promising solvents for electrospinning, enabling the production of high-quality nanometric fibers. Despite their potential, challenges persist in electrospinning polymer solutions containing ILs or poly­(ionic liquid)­s, mainly due to changes in solution conductivity, leading to rheological alterations and jet instabilities. In contrast, Solution Blown Spinning (SBS) offers a solution to these challenges, employing a pressurized air system without electric fields and ensuring a high rate of fiber production. This study aims to produce polymeric submicrometric fibers by leveraging the benefits of ILs as additives, along with the practicality and adaptability of the SBS technique. Twelve compositions of poly­(vinyl alcohol) (PVA) and poly­(vinylpyrrolidone) (PVP) using 2-hydroxydiethylammonium propionate (2-HDEAPr) PIL were spun and characterized by SEM, FTIR, and DSC analyses. The addition of 0.25 mL of PIL was sufficient to reduce the average diameter compared to pure fiber, due to the plasticizing effect of the additive. The findings confirm the feasibility of fiber production by SBS, highlighting fiber smoothness, alignment, and continuity. The average fiber diameters measured were 0.55, 0.66, and 0.91 μm, respectively, for PVA-0.25 mL, PVA-0.50 mL, and PVA-1 mL PIL. Additionally, PVP compositions exhibited an average diameter of approximately 0.40 and 0.55 μm. Such results indicate the potential of these aqueous solution blow-spun fibers for the development of eco-friendly fibrous materials.

## Graphical abstract

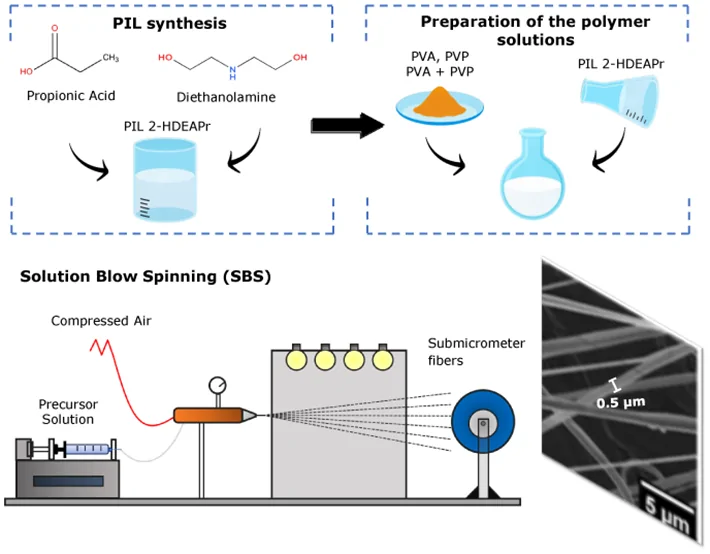

## 1 Introduction

The straightforward, simple, low-cost, and high-productivity solution blow spinning (SBS) technique has been widely used for micro- and nanofiber production.^1,2^ Among the applications are biomedical materials,^3,4^ high-performance filters,^5^ catalysis,^6^ and the production and storage of clean energy.^7,8^ Fibers in the nanometric range have been produced by SBS from a variety of polymers such as poly­(vinyl alcohol) (PVA), polyvinyl chloride (PVC), nylon-6, poly­(lactic acid) (PLA), poly­(3-hydroxybutyrate) (PHB), poly­(vinylpyrrolidone) (PVP), and poly­(D,L-lactic acid) (PDLLA).^9–12^ Additionally, aqueous SBS is an environmentally benign technique that reduces fiber production costs and prevents biomaterial contamination by toxic solvents.^13^

The most versatile methods for fiber production are melt-blowing, electrospinning (ES), supersonic solution blowing (SSB), and solution blow spinning (SBS). These techniques allow the adjustment of processing conditions, control of the average fiber diameter, and fabrication of fibrous materials from various polymers, both natural and synthetic.^2,14^ ES is the most employed fiber fabrication technique. However, when compared to SBS, it has some drawbacks such as a lower fiber throughput, higher equipment and spinning costs, and limitations regarding which solvents can be spun, including highly toxic ones such as dichloromethane, trifluoroethanol, tetrahydrofuran, *N*,*N*-dimethylformamide, or hexafluoro-2-propanol.^15–17^ The choice of solvent, used as a polymer spinning agent, is essential to the optimization of spun fibers by both ES and SBS. In both processes, the solvent type and concentration can affect solution spinnability and the morphology of the produced fibers.^18,19^

Ionic liquids (ILs) are a class of molten salts that, unlike traditional fluids, are composed entirely of ions and remain in the liquid state at temperatures near or below 100 °C. Their physicochemical properties can be tailored for specific applications through the appropriate combination of cation, anion, and alkyl chain.^20^ Due to their unique properties, such as adjustable organizational structure, biocompatibility, nonvolatility and nonflammability, variable solubility, high chemical and thermal stability, high ionic conductivity, and wide electrochemical potential window when compared to conventional organic solvents, ILs have been widely recognized as sustainable and promising materials, attracting great interest^21^ and finding applications in various fields, including metal separation and recovery,^22^ electrocatalyst synthesis,^23,24^ and as alternatives for several processes such as chemical synthesis, enzymatic catalysis, and applications in green engineering.^25^

Protic ionic liquids (PILs) are a subclass of ILs synthesized through a simple neutralization reaction involving the proton transfer from a Brønsted acid to a Brønsted base. The interactions between cations and anions consist of a combination of hydrogen bonding, Coulombic charge interactions, and dispersion forces.^26–28^ The key feature governing the behavior of ILs is the formation of a hydrogen-bonded structure resulting from proton exchange. These hydrogen bonds impart unique properties to PILs, such as enhanced solubility, tunable viscosity, and the ability to participate in specific chemical interactions, making them highly adaptable for a wide range of applications. This subclass also offers additional advantages due to its simple synthesis, low cost, biodegradability, and often lower toxicity.^29^

Recent advances across various scientific and industrial fields have driven the potential applications of PILs. In sustainable chemistry, PILs have been extensively explored as electrolytes for electrodeposition due to their wider electrochemical windows and ability to enable the deposition of water-sensitive compounds.^28^ Another remarkable development involves their application in proton exchange membranes.^30^ The intrinsic properties of PILs, such as high thermal stability and proton conductivity at elevated temperatures (>100 °C), allow fuel cell operation under anhydrous conditions.^27^ Moreover, significant research efforts have focused on the thermophysical characterization of PILs, analyzing properties such as density, viscosity, and conductivity to optimize their performance in various industrial processes, including gas separation and as electrolytes in lithium batteries and other electrochemical devices. Further applications and in-depth discussions regarding the behavior of PILs can be found in the literature.^31–33^

ILs have been tested as solvents for fiber fabrication by electrospinning,^34–36^ producing nanometric-sized and defect-free fibers. The fact that ILs are nonvolatile and nonflammable is an advantage over many other solvents. However, spinning polymer solutions containing ILs or poly­(ionic liquid)­s is still difficult by electrospinning.^37^ This is due to several problems affected by fluctuations in the solution conductivity, causing rheological changes and jet instabilities, particularly when processing highly polar or aqueous formulations. On the other hand, since SBS is a technique based on a pressurized air system that does not require the use of electric fields, it exhibits high tolerance to variations in solvent polarity or solution conductivity, allowing a wide range of polymeric solutions to be spun.^13,38^

ILs have demonstrated significant potential in environmentally and biologically relevant applications due to the synergistic combination of polymer processability and functionality. In the context of wastewater treatment, Rosli et al. developed electrospun chitosan/PVA nanofibers functionalized with an imidazolium-based ionic liquid, which exhibited markedly enhanced adsorption performance toward heavy metal ions, particularly Pb­(II), achieving adsorption capacities up to 166.34 mg g^–1^ and demonstrating the effectiveness of IL functionalization in improving adsorption efficiency and water stability.^39^ Additionally, polymer–IL-based fibrous materials have been explored for antimicrobial applications, as demonstrated by Libel et al., who reported that IL-containing polymeric fibers exhibited significant antimicrobial activity attributed to the presence of ionic moieties, reinforcing their suitability for bioactive surfaces and protective materials.^40^ Collectively, these studies illustrate that polymer–IL-based fibrous systems can be rationally designed to address these challenges with strong potential for sustainable and functional material development.

The choice of the PIL 2-hydroxydiethylammonium propionate (2-HDEAPr) was motivated by a combination of environmental, economic, and processing considerations. Unlike many aprotic ionic liquids based on imidazolium or pyridinium cations, which often require multistep syntheses, halogenated reagents, and extensive purification, 2-HDEAPr is obtained through a straightforward acid–base neutralization between diethanolamine and propionic acid. Both precursors are low-cost, commercially available, and commonly employed in industrial and laboratory applications, which significantly reduces the overall environmental footprint and economic cost of the IL.^41^

From a processing standpoint, the 2-HDEAPr PIL exhibits complete miscibility with water, moderate viscosity, and strong hydrogen-bonding capability, enabling efficient solvation of hydrophilic polymers such as PVA and PVP. These characteristics are particularly advantageous for aqueous SBS, as they mitigate limitations commonly encountered in the electrospinning of IL-containing solutions, such as excessive conductivity and jet instability. Moreover, when compared to conventional volatile organic solvents typically used for fiber spinning (e.g., DMF, HFIP, or TFE), 2-HDEAPr is nonvolatile, nonflammable, and allows fiber production under safer and more sustainable conditions.^42^

This study focused on the synthesis and characterization of PVA/PVP polymeric fibers utilizing 2-HDEAPr PIL as part of the aqueous solvent system produced via the SBS technique. The choice for PVP and PVA was motivated by their extensive versatility and widespread use in the production of micro- and nanofibrous membranes, serving both as functional polymeric fibers and as templates for metal oxide hollow structures. Despite the well-established use of ILs in nanofiber production by electrospinning, it is still scarce in SBS, which is a simple, cost-effective, and scalable technique.^1,2^ For this purpose, we systematically and integratively investigated the effect of PIL concentration on the rheological properties of the precursor solutions and on the morphological, chemical, and thermal characteristics of the resulting fibers, enabling their application in areas such as tissue engineering, biomedical scaffolds, and biodegradable film technologies.

Furthermore, SBS enables the production of spun membranes by combining polymers and ILs for applications that include solid electrolytes for batteries, proton-conducting membranes in fuel cells, biomaterials, as well as advanced sensors and biosensors, paving the way for innovation in energy storage, biomedical, and environmental technologies.

## 2 Experimental Section

### 2.1 Protic Ionic Liquid Synthesis

The amine compound (diethanolamine, Merck Synthesis, 99%) was placed in a three-necked flask glassware assembly equipped with a reflux condenser, a PT-100 temperature sensor for controlling temperature, and a dropping funnel. The flask was mounted in a thermal bath. Heating to approximately 50 °C was required to enhance the miscibility of the reactants and enable the reaction to proceed. The organic acid (propionic acid, Merck Synthesis, 99%) was added dropwise to the flask under stirring with a magnetic bar. Stirring continued for 24 h at room temperature (22 °C) to obtain a final viscous liquid. No solid crystals or precipitation was noted when the liquid sample was purified or stored in a freezer (−5 °C) for a few months after synthesis.

### 2.2 Preparation of the Polymer Solutions and Spinning by SBS

Twelve (12) compositions were based on a simple and systematic experimental design approach. Since only two hydrophilic polymers were investigated—poly­(vinyl alcohol) (PVA) and polyvinylpyrrolidone (PVP)—we defined three main solution systems: (i) pure PVA, (ii) pure PVP, and (iii) a PVA/PVP blend (1:1). For each of these three base polymer solutions, four different concentrations of the PILs (Figure 1)—0, 0.25, 0.50, and 1.00 mL per 5 mL of polymer solution—were selected. First, solutions of PVA (Mw = 49,000 g·mol^–1^, ρ ≈ 1.26–1.31 g·cm^–3^, *T*_g_ ≈ 75–85 °C (dry), *T*_m_ ≈ 220–230 °C, from Kuraray) and PVP (Mw = 1,300,000 g·mol^–1^, ρ ≈ 1.19–1.22 g·cm^–3^ (dry), *T*_g_ ≈ 150–180 °C, from Sigma-Aldrich) were prepared by dissolution in distilled water. PVA was dissolved at 16% (w/v) at 90 °C overnight under constant stirring, and PVP was dissolved at 10% (w/v) under vigorous stirring at room temperature (22 °C).^43^ One blended solution of PVA/PVP was also prepared using a mixture with 2.5 mL of each of the above solutions in a PVA/PVP ratio of 1:1. Then different proportions of the PIL were added for later spinning, where 1 mL of PIL was used as the maximum volume. Table 1 details the developed compositions.

**Figure 1:**
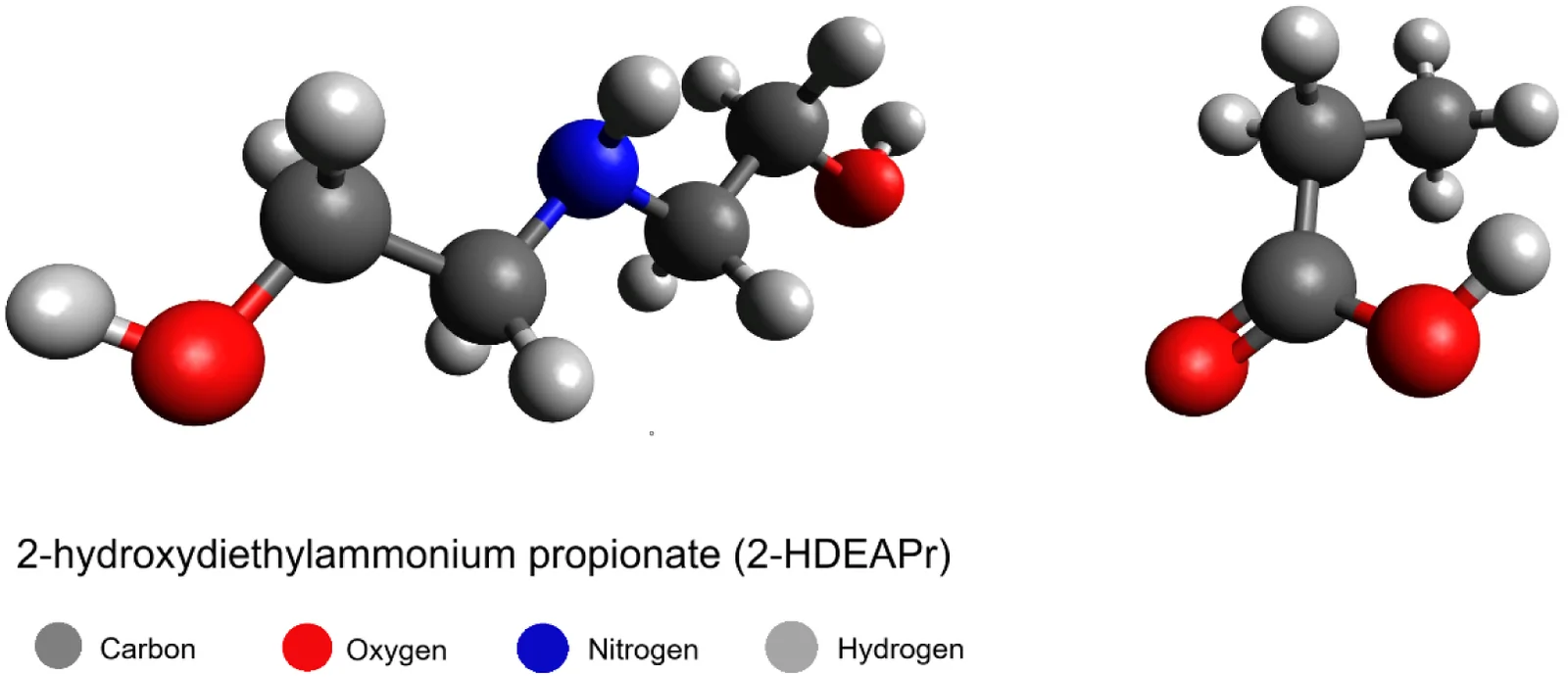
Three-dimensional representations of the 2-hydroxydiethylammonium (cation) and propionate (anion) ions that constitute the 2-HDEAPr PIL (created by authors with Avogadro).

**Table 1:** Compositions Used for Fiber Production by SBS from PIL^a^

Polymer solution and blend	Concentration (% w/v)	PIL added in 5 mL of PS and blend (mL)	PIL content relative to PS (% v/v)	PS:PIL molar ratio^b^
PVA	16	0.00	Pure PVA	1:0
PVA	16	0.25	5	11.47:1
PVA	16	0.50	10	5.74:1
PVA	16	1.00	20	2.87:1
PVP	10	0.00	Pure PVP	1:0
PVP	10	0.25	5	2.84:1
PVP	10	0.50	10	1.42:1
PVP	10	1.00	20	0.71:1
PVA + PVP (1:1 v/v)	13^c^	0.00	PVA/PVP blend	1:0
PVA + PVP (1:1 v/v)	13^c^	0.25	5	7.16:1
PVA + PVP (1:1 v/v)	13^c^	0.50	10	3.58:1
PVA + PVP (1:1 v/v)	13^c^	1.00	20	1.79:1

a PS = polymeric solution; PIL = protic ionic liquid.

b Molar ratio calculated based on the molar mass (M ≈ 179.22 g·mol^–1^) and density (ρ ≈ 1.134793 g.cm^–3^) of 2-hydroxy diethylammonium propionate at 25 °C.^42^

c Total polymer concentration in the blend (8% (w/v) PVA + 5% (w/v) PVP) due to mutual dilution upon mixing equal volumes of stock solutions.

The experimental setup of the SBS process is illustrated in Figure 2, based on a pressurized air flow that carries the precursor solution and pumps it through a hot stage for rapid solvent evaporation and fiber collection at the collector. All fibers were produced using SBS with a hot stage (zone) to force a quick water evaporation, as previously reported.^13,43^ The SBS process was conducted using a flow rate of 2.5 mL·h^–1^, air pressure of 60 psi, and ambient humidity ranging from 40–50%. The nozzle inner and outer diameters were 1.5 and 5 mm, respectively, while the protrusion length was 15 mm for both polymers.^13,43^ The nozzle-to-collector working distance was 60 cm, while the hot stage was 40 cm and kept constantly heated to ≈45 °C. A rigid cylindrical aluminum collector was used, rotating at 150 rpm.

**Figure 2:**
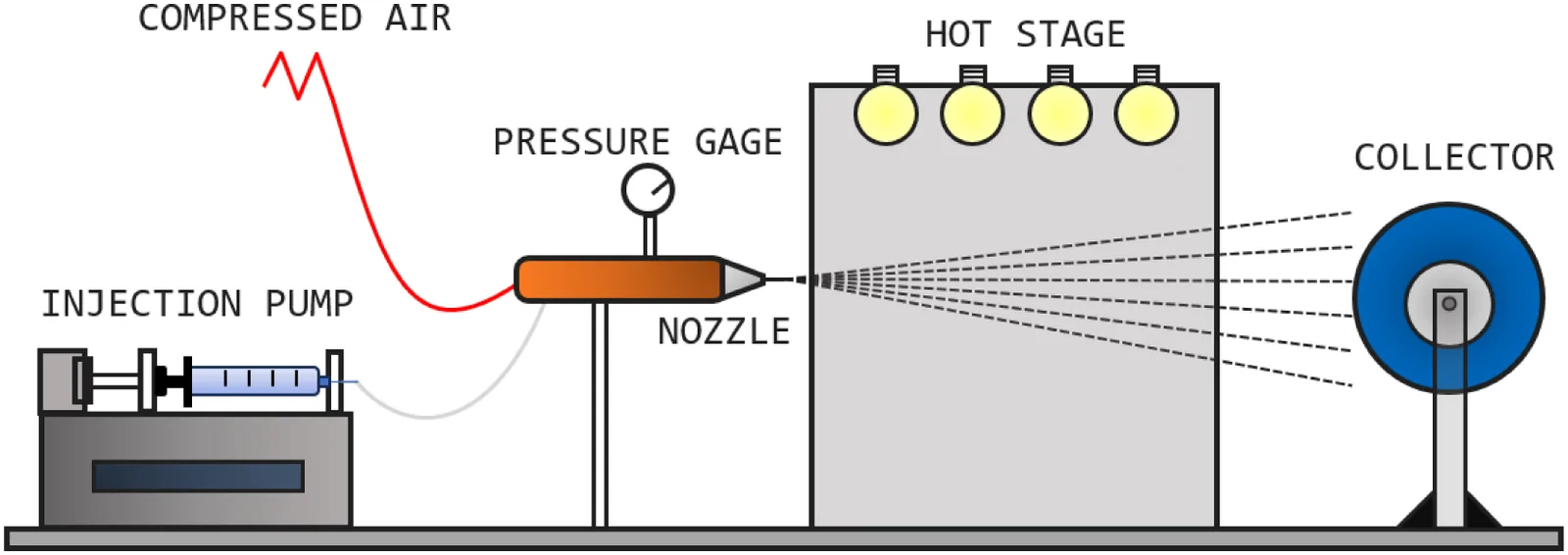
Schematic of the heated SBS apparatus used for aqueous polymeric fiber production.

### 2.3 PIL and Polymeric Solution Characterization

The rheological properties of the solutions were characterized by means of a Brookfield Rheometer DV-III Ultra (0–100 rpm) equipped with a thermostatic bath for precise temperature control. Measurements were recorded after the readings reached equilibrium. The measurements were conducted using a CP-52 cone geometry for 0.50 mL of solution and a cone angle of 3° at 25 °C.

### 2.4 Fiber Characterization

Fiber morphology was evaluated by Scanning Electron Microscopy (SEM) using LEO 1430 Zeiss equipment. Prior to analysis, fibers were gold-coated using an Emitech K550X metallizer. Images were acquired in secondary electron mode using a working distance of 8.4 mm and an accelerating voltage of 5.0 kV. The obtained images were then used to measure the average diameter from at least 50 fibers using ImageJ software, developed by the National Institutes of Health in the USA. The diameters of the fibers produced were analyzed using a box-and-whisker diagram, which allows us to visually estimate various L-estimators, notably the interquartile range, midhinge, midrange, and trimean. Quantitative data were analyzed using the Shapiro–Wilk test for normality of residuals and the Brown–Forsythe test for evaluation of homoscedasticity. The Kruskal–Wallis test (α = 0.05) followed by Dunn’s post hoc test was applied to determine significant differences among the experimental groups (*p* < 0.05), and results were presented in the Supporting Information (Figures S1 and S2).^44,45^

Infrared spectra were obtained by Fourier transform infrared spectroscopy (FTIR), recorded on a PerkinElmer 400 spectrometer, using potassium bromide (KBr) tablets in the range of 400 and 4000 cm^–1^ with a resolution of 4 cm^–1^ by averaging 40 scans. Differential scanning calorimetry (DSC) was performed by using a model DSC-60 Plus thermal analyzer from Shimadzu. Samples were heated from 25 to 300 °C at a heating rate of 20 °C/min under a nitrogen gas stream (50 mL·min^–1^).

## 3 Results

Figure 3 presents the rheological characterization of the pure polymers and the PIL, as well as PVA, PVP, and PVA/PVP blend solutions containing 2-HDEAPr PIL. As shown in Figure 3a, the 2-HDEAPr PIL exhibits non-Newtonian behavior over specific shear rate ranges, consistent with previous reports for ionic liquids.^41,46^ Specifically, an initial shear-thickening response is observed at low shear rates (0–15 s^–1^), where the viscosity increases from approximately 0.110 to 0.126 Pa·s. Subsequently, in the intermediate shear rate region (20–100 s^–1^), the viscosity remains nearly constant, with only minor fluctuations around 0.125–0.127 Pa·s.

**Figure 3:**
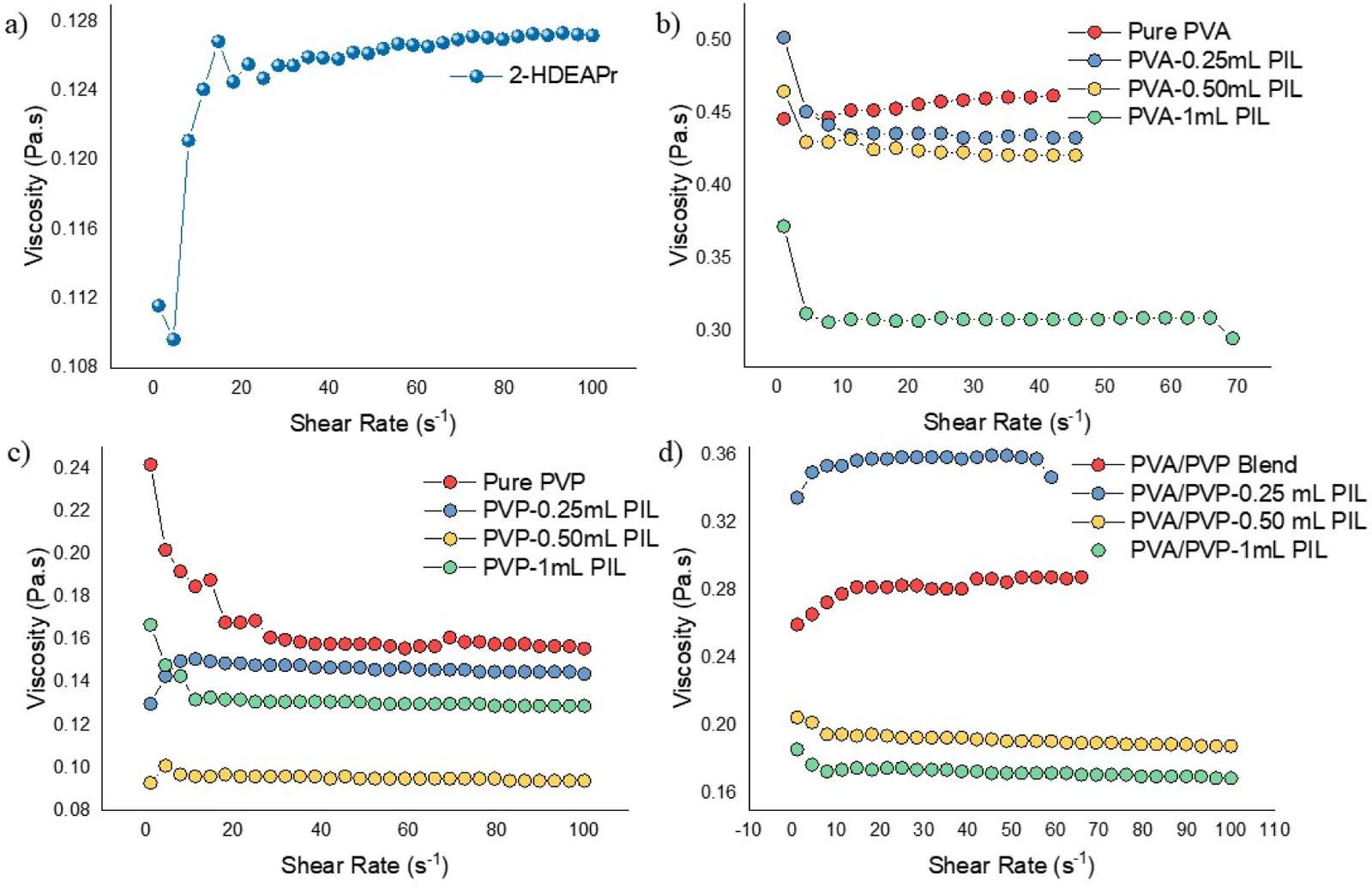
Viscosity–shear rate curves for (a) 2-HDEAPr PIL, (b) PVA system, (c) PVP system, and (d) PVA/PVP blend with different PIL contents.

The rheological behavior of the 2-HDEAPr PIL reveals a highly structured liquid whose initial viscosity is influenced by strong intermolecular interactions and long-range collective mechanisms.^47^ The initial shear-thickening tendency at low shear rates, followed by a regime of relatively constant viscosity at higher shear rates, suggests that PILs exhibit dynamic molecular structuring. In this system, ionic and hydrogen-bonding interactions between the cation and anion form a transient network that becomes stabilized under higher shear conditions. Therefore, the shear-thickening behavior of the PIL, combined with the shear-thinning behavior of PVA,^48^ for instance, results in a solution with controlled viscosity, which may enhance jet stability and elongation during the fiber-fabrication process.

The rheological behavior of the solutions varied as a function of polymer composition and the concentration of the added PIL. In general, the solutions exhibited predominantly Newtonian behavior over the entire shear rate range investigated. However, for pure PVP, a slight pseudoplastic tendency was identified (n slightly lower than 1), though without significant rheological relevance within the studied shear range. The viscosity values ranged from a maximum of 0.46 Pa·s to a minimum of 0.44 Pa·s for PVA, from 0.16 to 0.24 Pa·s for PVP, and from 0.26 to 0.29 Pa·s for the PVA/PVP blend.

The addition of PIL generally promoted a reduction in viscosity with increasing IL concentration, except for the PVA/PVP blend at the concentration of 0.25 mL, which exhibited an initial viscosity enhancement; however, the overall flow behavior of the solutions remained Newtonian. This suggests a predominantly plasticizing effect of the IL,^49^ acting mainly as a disrupting agent of the intermolecular network and enhancing polymer chain mobility.^40^ These findings are particularly relevant for the SBS process since solution viscosity directly influences jet stability and the final fiber diameter. The decrease in viscosity, together with other effects induced by the PIL reported in the literature,^50,51^ may favor greater jet stretching, thereby explaining the experimentally observed trend toward smaller fiber diameters. Nevertheless, at higher concentrations, additional rheological effects may modify the tendency.

Na et al. concluded that the incorporation of the investigated ionic liquids significantly increased the electrical conductivity while simultaneously decreasing the viscosity of the PLA (polylactide) solution. This combined effect influenced the stretching behavior of the solution during electrospinning and, consequently, led to a reduction in the diameter of the electrospun fibers.^51^ Libel et al. emphasized in their studies that ILs may play a dual role as both plasticizers and surfactants in polymer solutions. At low concentrations, ILs can significantly reduce the viscosity of polymeric systems. Acting as plasticizers, ILs attenuate interactions between polymer–polymer chains, decreasing polymer entanglements and lowering the viscosity. This reduction in viscosity may also contribute to a decrease in the surface tension.^40^

The morphology of the produced fibers was observed by SEM. Figure 4 shows a high-magnification image, while Figure S3 in the Supporting Information shows a low-magnification image. The influence of the 2-HDEAPr PIL on the morphology of the solution blow-spun fibers was evaluated by comparison with fibers without any additive. Pure PVA exhibited smooth, parallel fibers with an ordered and regular alignment, free of apparent visual defects like casting or beads. Due to the aliphatic structure of PVA, the organization enables the formation of a well-defined fiber membrane.^37,52^ Pure PVP fibers formed relatively disordered networks, a less regular structure, and some fibers presented a mildly curved morphology. The fibers formed by the PVA/PVP blend promoted a denser and more heterogeneous mat with the appearance of surface irregularities. In addition to the main fibers with micrometer diameters, the presence of thinner secondary fibers can be observed, distributed among the thicker filaments. These structures exhibit a noticeably smaller diameter, forming a complementary mat that fills the voids within the fibrous network.

**Figure 4:**
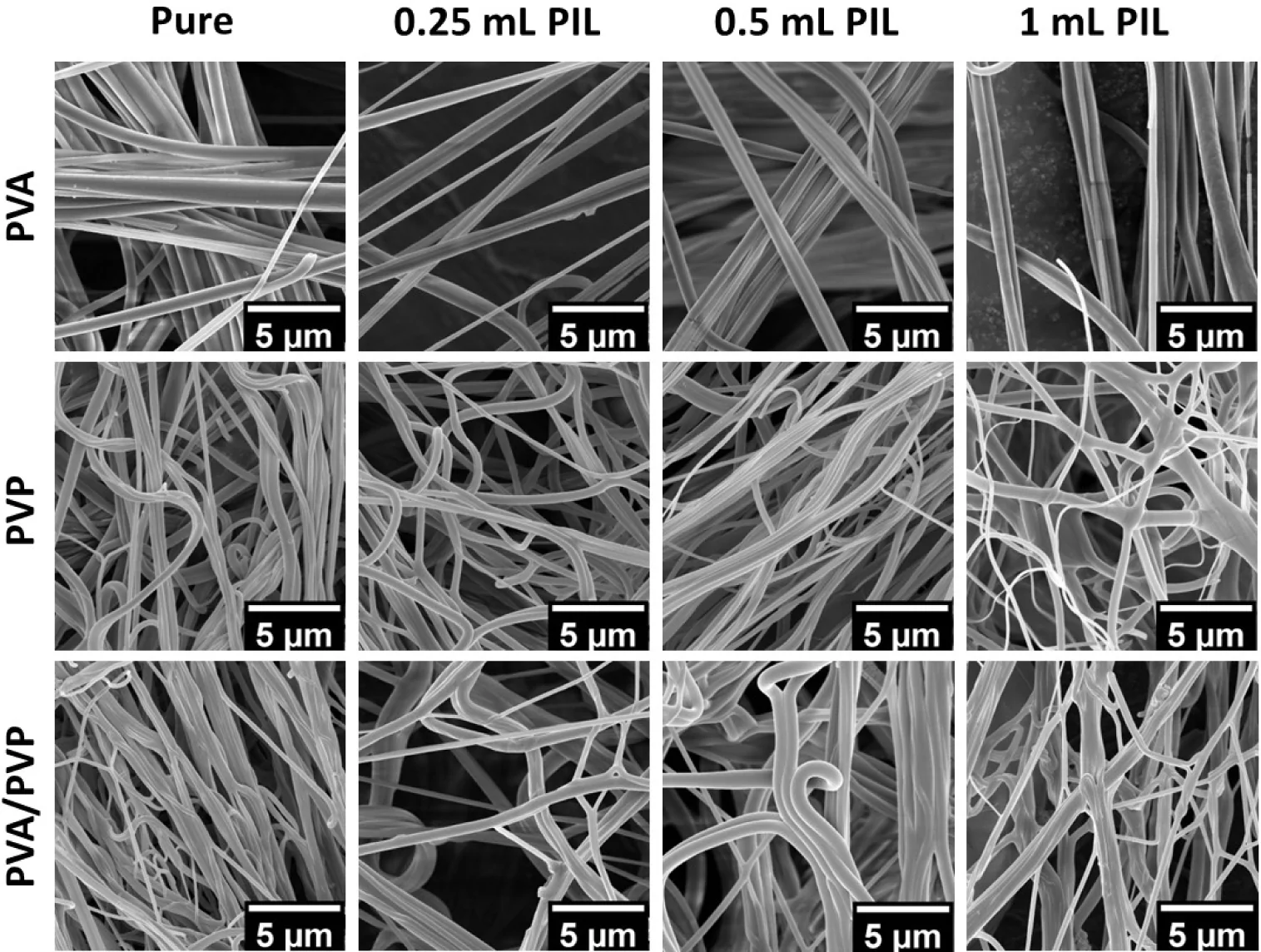
SEM micrographs of spun fibers.

The incorporation of PILs significantly influences fiber morphology, leading to noticeable changes in diameter distribution, surface features, and overall network organization. For PVA, the addition of a small amount of the PIL 2-HDEAPr was sufficient to cause a drastic reduction in the average diameter of the fibers, as well as to render them more homogeneous and uniform. For pure fibers, the average diameter was 1.12 μm, whereas with the addition of only 0.25 mL of PIL, this value decreased to 0.55 μm. For PVP and the PVA/PVP blend, the addition of PIL resulted in the formation of more interlaced and heterogeneous fibrous networks. Different structures can be observed, characterized by the coexistence of micrometric fibers and thinner filaments distributed among them. Intersection points between fibers are particularly evident in the SEM images of PVP-0.25 mL PIL and PVA/PVP-1 mL PIL, which may have undergone slight fusion, resulting in branched fibers. The surface of the PVA fibers remained relatively uniform, indicating that solidification occurred to a reasonable extent before reaching the collector. For the PVP fibers and the PVA/PVP blend, the observed junctions and branching are associated with incomplete evaporation of water and the PIL. The results obtained are consistent with previous observations reported also for electrospun fibers.^13,53^ A plausible hypothesis for the changes observed in fiber diameter, orientation, entanglement, and junction or fusion points, as evidenced by the SEM micrographs, is the increased water retention and wettability of the system with increasing PIL concentration. This effect hinders the complete evaporation of water during the spinning process. As a result, the fibers reach the collector while still partially wet, promoting conjugated fibers, which increase in the final fiber diameter. This has already been observed in aqueous systems by SBS.^13,54^ As noted, the morphology and diameter of fibers produced by SBS are influenced by variables related to the polymer solution, and consequently to the solvent used, as well as by intrinsic process parameters.

The measurements of the diameter size distributions, shown in Figure 5 as box–whisker plots, highlight the stability of the fibers produced under different conditions with the addition of the 2-HDEAPr PIL. It is possible to notice that increasing the 2-HDEAPr PIL content in PVA from 0.25 to 1 mL promotes fiber smoothness, alignment, and continuity. The diameter distribution values, represented by the interquartile range (IQR) in the box plot, vary approximately between 0.40 and 0.80 μm, indicating that 50% of the fibers fall within this range, with an average diameter of 0.55 ± 0.23 μm for PVA-0.25 mL PIL, and evidencing a significant 50.71% reduction in the average fiber diameter compared to the additive-free sample (1.12 ± 0.66 μm). For PVA-0.50 mL PIL, an average diameter of 0.66 ± 0.36 μm was obtained, and for PVA-1 mL PIL, the IQR expanded from 0.70 to 1.10 μm, with a mean diameter of 0.91 ± 0.34 μm.

**Figure 5:**
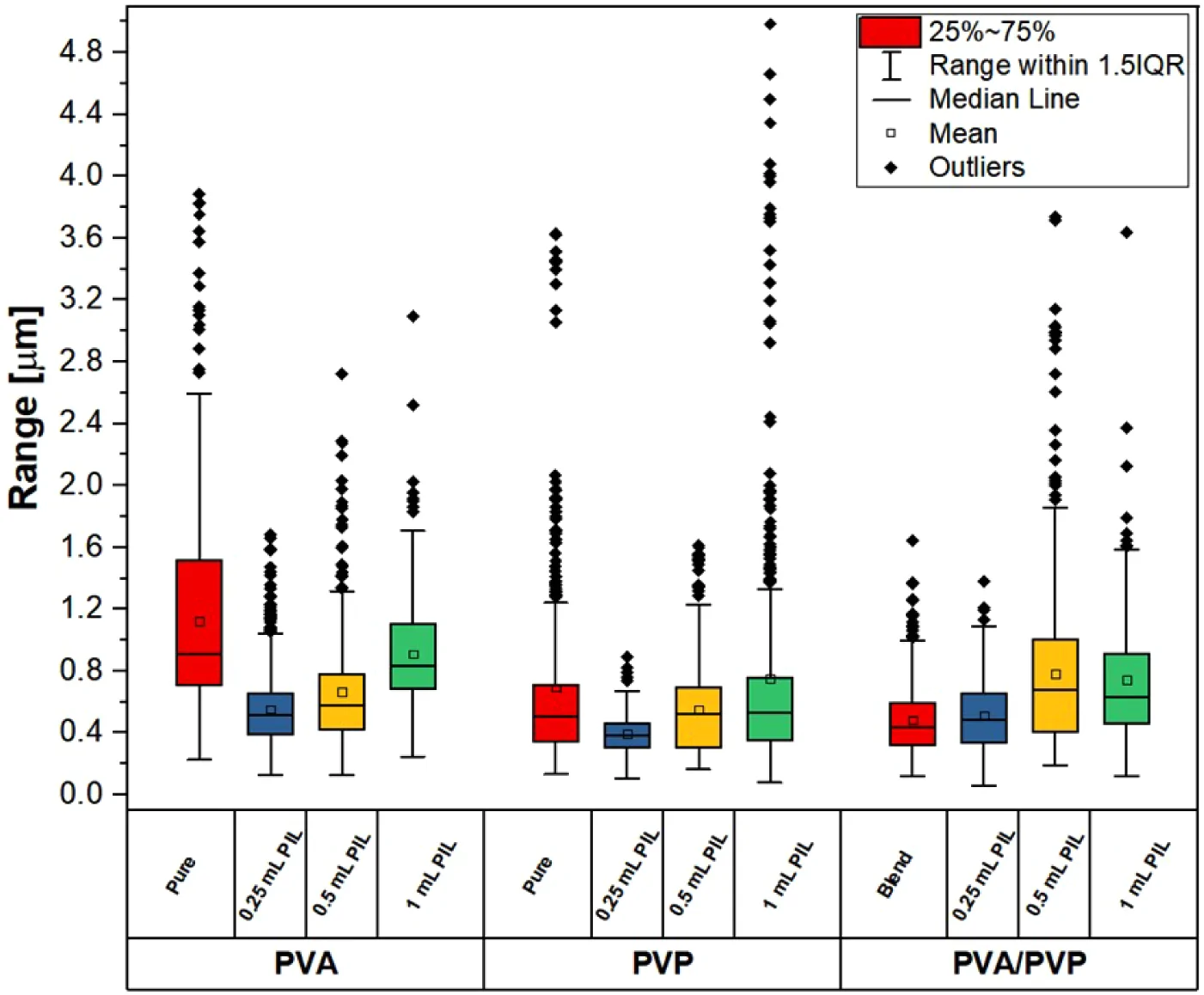
Diameter distribution of spun fibers obtained by SBS of PVA, PVP, and PVA/PVP with different concentrations of 2-HDEAPr PIL.

The SEM image of the PVP fibers reveals a distinct morphology characterized by disorganized fibers and the formation of an interconnected network structure with increasing PIL content, likely resulting from the high molecular weight of PVP, which promotes steric hindrance and hinders uniform jet stretching during fiber formation. When the PIL amount is added to the solution, the formation of this structure occurs, as observed in the literature.^55^

Regarding the PVP-based system, the addition of PIL induced a significant narrowing of the fiber diameter distribution, leading to lower dispersion, particularly for the 0.25 mL incorporation. For the pure PVP sample, a broad distribution with high variability was observed, yielding a mean diameter of 0.69 ± 0.88 μm. However, a significant diameter reduction was achieved when a small quantity of 0.25 mL of PIL was added to the solution, resulting in a 42.69% reduction in the average fiber diameter, which dropped to 0.40 ± 0.14 μm. This enhanced microstructural homogeneity is evidenced by its highly compact interquartile range (IQR), which spans tightly from approximately 0.28 to 0.45 μm. As the PIL content increased to 0.5 mL, the average diameter slightly increased to 0.55 ± 0.29 μm. Finally, for the PVP-1 mL PIL sample, the mean diameter reached 0.75 ± 0.89 μm, accompanied by a wider distribution range and higher dispersion.

At low concentrations, the PIL exerts a similar plasticizing effect on PVP as previously observed for the PVA matrix, significantly decreasing fiber dimensions. However, increasing the PIL content beyond 0.25 mL led to a progressive increase in both the average diameter and distribution range for most compositions. Thus, for both pure polymeric matrices, the incorporation of 0.25 mL of PIL was identified as the optimal concentration for achieving the thinnest and most homogeneous fiber diameters. When compared to the PVA/PVP blend, that was not observed because the complex interactions between the polymer’s chains and PIL, added to the PVP high molecular weight, increased the solution viscosity, mainly for the 0.25 mL IL content.^56^ The variation in fiber diameter with increasing PIL content showed a nonlinear trend for PVA, PVP, and the PVA/PVP blend. For PVA and PVP, the initial addition of PIL promoted a marked reduction in the average fiber diameter, likely due to the plasticization effect of PIL and decreased viscosity, which possibly intensified jet stretching during spinning. In contrast, for the PVA/PVP blend, although an initial diameter reduction was also observed, further increases in PIL content did not lead to a proportional decrease. At higher concentrations, changes in viscoelastic behavior^50^ and polymer–PIL interactions^40^ likely altered chain entanglement density and decreased the stretching, thereby promoting the formation of fibers with larger diameters. These findings indicate that fiber diameter is governed by a complex balance between rheological properties and molecular interactions, rather than by a simple linear dependence on PIL concentration.

To evaluate the effect of PIL incorporation on the average diameter of the fibers obtained by SBS, the Shapiro–Wilk test for normality of residuals and Brown–Forsythe test (Figure S1) for evaluating homoscedasticity were initially performed at a 5% significance level. For all evaluated conditions, the test categorically rejected the hypothesis of normality for the distributions (*p* < 0.05), and, at the 0.05 level, it was observed that the population variances are significantly different. Given the nonparametric nature of the experimental data, the statistical significance of the morphological changes was determined using the Kruskal–Wallis test followed by Dunn’s post hoc test. The analysis revealed that the addition of PIL exerted a highly significant effect on the fiber diameter across all studied systems (Figure S2), driving mathematically a clear distinction between the pure polymers and the modified systems by PIL.

The analysis of the population medians indicated distinct spinning behaviors between the pure polymers and the blend. For the pure PVA and PVP, the lowest PIL addition (0.25 mL) induced a drastic reduction in fiber diameter, with medians decreasing from 0.92 to 0.52 μm for PVA and from 0.51 to 0.38 μm for PVP. Higher PIL content (1.00 mL) caused an opposite effect, raising the median diameters to levels close to or slightly higher than the pure controls, indicating the optimal results for decreases. This is further corroborated by the absence of a statistically significant difference, as indicated by Sig = 0 in Dunn’s test, between the groups with higher PIL concentrations (1.00 mL) and their respective pure controls (*p* = 1.000). Conversely, the PVA/PVP blend exhibited a significantly smaller median diameter (0.33 μm) than both pure polymers. Interestingly, the impact of PIL incorporation on the blend followed a trend inverse to that of the pure polymers: concentrations of 0.25 and 0.50 mL increased the median diameters to 0.48 and 0.68 μm, respectively. This anomalous behavior is directly governed by the solution’s rheological properties. As evidenced in Figure 3d, the PVA/PVP-0.25 mL solution exhibited the highest shear viscosity among all tested systems. Particularly in groups with higher concentrations of PIL, the presence of fiber diameters exceeding 3.0 μm suggests that alterations in rheological properties and the delay in solvent evaporation influence the additional thinning that may occur at the collector following fiber fusion.^13,57^ Consequently, the deposition of fibers in a plastic state hinders final stretching and promotes coalescence into larger diameter structures, which constitute the observed outliers in the distribution.

Table 2 summarizes the fiber diameters obtained using different ILs via various spinning techniques; however, no studies using solution blow spinning (SBS) were found, except for the work of Zhang et al., which employed an adapted SBS approach. While conventional SBS relies on solvent evaporation to solidify the fiber, the system in this study employs water mist-induced coagulation applied during the blowing process.^58^ A nonlinear trend in fiber diameter was also observed. Libel et al. employed PVA and acetic acid to fabricate adhesive fibers with antimicrobial properties, achieving an average fiber diameter of 0.31 μm. The incorporation of C_16_MImCl (1-Hexadecyl-3-methylimidazolium chloride) led to a slight reduction in diameter to 0.29 μm, which was attributed to the decreased surface tension of the solution upon IL addition.^40^ Asai et al. investigated the effect of different ILs on the fabrication of PVDF (poly­(vinylidene fluoride)) fibers. The addition of ILs exerted a significant influence on the fiber diameter, although the behavior was not strictly linear. The authors attributed the formation of finer fibers, among other factors, to the charge repulsion introduced by the incorporation of the IL.^55^ Zhang et al. produced fibers via wet-type solution blow spinning, where the polymer jet is attenuated by high-velocity hot air and regenerated in a water-mist coagulation chamber. Under optimized conditions, uniform submicrometer fibers with an average diameter of 0.98 ± 0.62 μm were obtained, forming homogeneous nonwoven mats. Regenerated cellulose obtained from a high-degree of polymerization dissolving pulp (DP ≈ 2365) was used as the polymer matrix to ensure stable fiber formation. The cellulose was dissolved in the nonvolatile ionic liquid 1-ethyl-3-methylimidazolium diethyl phosphate (EMIMDEP), which provides adequate rheological control without significant polymer degradation.^56^

**Table 2:** Effect of Different Ionic Liquids on the Diameter of Fibers Produced by Electrospinning Techniques^a^

Polymer	IL	⌀ Initial (nm)	⌀ Final (nm)	IL Function	Ref
PVA	HMIMCl	118 ±19 (0.0 mol IL)	132 ± 23 (0.04 mol IL)	Conductive additive	^57^
			153 ± 33 (0.08 mol IL)		
			113 ± 23 (0.25 mol IL)		
Cellulose	[C_2_mim][CH_3_CO_2_]/ [C_10_mim]Cl	470 ± 110 nm (1 mol [C_2_mim][CH_3_CO_2_])	120 ± 55 (0.90:0.10 mol IL)	- [C_2_mim][CH_3_CO_2_]: polymer dissolution	^59^
				- [C_10_mim]Cl: reduction of surface tension	
PVP-TiO_2_ NPs	[PEVImBr]/[NTf_2_^–^]	170 ± 21 (90:10 wt %)	93 ± 38 (80:20 wt %)	Polymer dissolution	^60^
PLLA	[EMIm][Cl]	1157 ± 147 (0% IL)	164 ± 63 ([EMIm][Cl])	Fiber properties modulation	^53^
	[EMIm][BF_4_]		193 ± 43 ([EMIm][BF_4_])		
	[EMIm][HSO_4_]		98 ± 24 ([EMIm][HSO_4_])		
	[EMIm][NTf_2_]		166 ± 44 [EMIm][NTf_2_]		
	[EMIm][OAc]		209 ± 75 [EMIm][OAc]		
	[EMIm][PF_6_]		167 ± 50 (1% [EMIm][PF_6_])		
			142 ± 35 (1.5% [EMIm][PF_6_])		
			174 ± 37 (2% [EMIm][PF_6_])		
			172 ± 42 (8% [EMIm][PF_6_])		
PLA	[EMIM]NTf_2_	395 ± 151 nm (0% IL)	117 ± 46 nm (5% IL)	Fiber properties modulation	^51^
			110 ± 41 (10% IL)		
			72 ± 34 (20% IL)		
			69 ± 32 (40% IL)		
PVDF	BMIMCl	241 ± 12 (0% IL)	181 ± 9 (3% IL)	Filler–conductive additive	^61^
			148 ± 7 (5% IL)		
			230 ± 11 nm (10% IL)		
CA	BMIMCl	125 (0% IL)	150 (2% IL)	Fiber properties modulation	^50^
			525 (6% IL)		
			180 (12% IL)		
SAN + 1,2 DCE	BMIMCl	2.000 (0% IL)	≅550 (5% IL)	Polymer dissolution	^62^
			≅250 (18.8% IL)		

a HMIMCl—1-Hexyl-3-methylimidazolium chloride; [C_2_mim]­[CH_3_CO_2_]—1-Ethyl-3-methylimidazolium acetate; [C_10_mim]­Cl—1-Decyl-3-methylimidazolium chloride; NPs—Nanoparticles; PEVImBr—Poly­(1-Ethyl-3-vinylimidazolium) bromide; (NTf_2_^–^)—Bis­(trifluoromethylsulfonyl)­imide; PLLA—Poly­(L-lactic acid); [EMIm]­[Cl]—1-Ethyl-3-methylimidazolium chloride; [EMIm]­[BF_4_]—1-Ethyl-3-methylimidazolium tetrafluoroborate; [EMIm]­[HSO_4_]—1-Ethyl-3-methylimidazolium hydrogen sulfate; [EMIm]­[NTf_2_]—1-Ethyl-3-methylimidazolium bis­(trifluoromethylsulfonyl)­imide; [EMIm]­[OAc]—1-Ethyl-3-methylimidazolium acetate; [EMIm]­[PF_6_]—1-Ethyl-3-methylimidazolium hexafluorophosphate; PLA—Polylactide; PVDF—Poly­(vinylidene fluoride); BMIMCl—1-Butyl-3-methylimidazolium chloride; CA—Cellulose acetate; SAN—Styrene-Acrylonitrile Copolymer; 1,2 DCE—1,2-Dichloroethylene.

The influence of 2-HDEAPr PIL addition on the polymer fibers was evaluated by FTIR. Spectra were collected for the 2-HDEAPr PIL in its liquid state, while all polymer-based samples (PVA, PVP, and PVA/PVP blends with varying PIL contents) were analyzed after spinning in the form of solid fibers. Upon mixing the components, the intermolecular interactions within the fibers are reflected by shifts in the characteristic bands. Figure 6 displays the infrared spectra of the pure components and their respective blends following the addition of the PIL.

**Figure 6:**
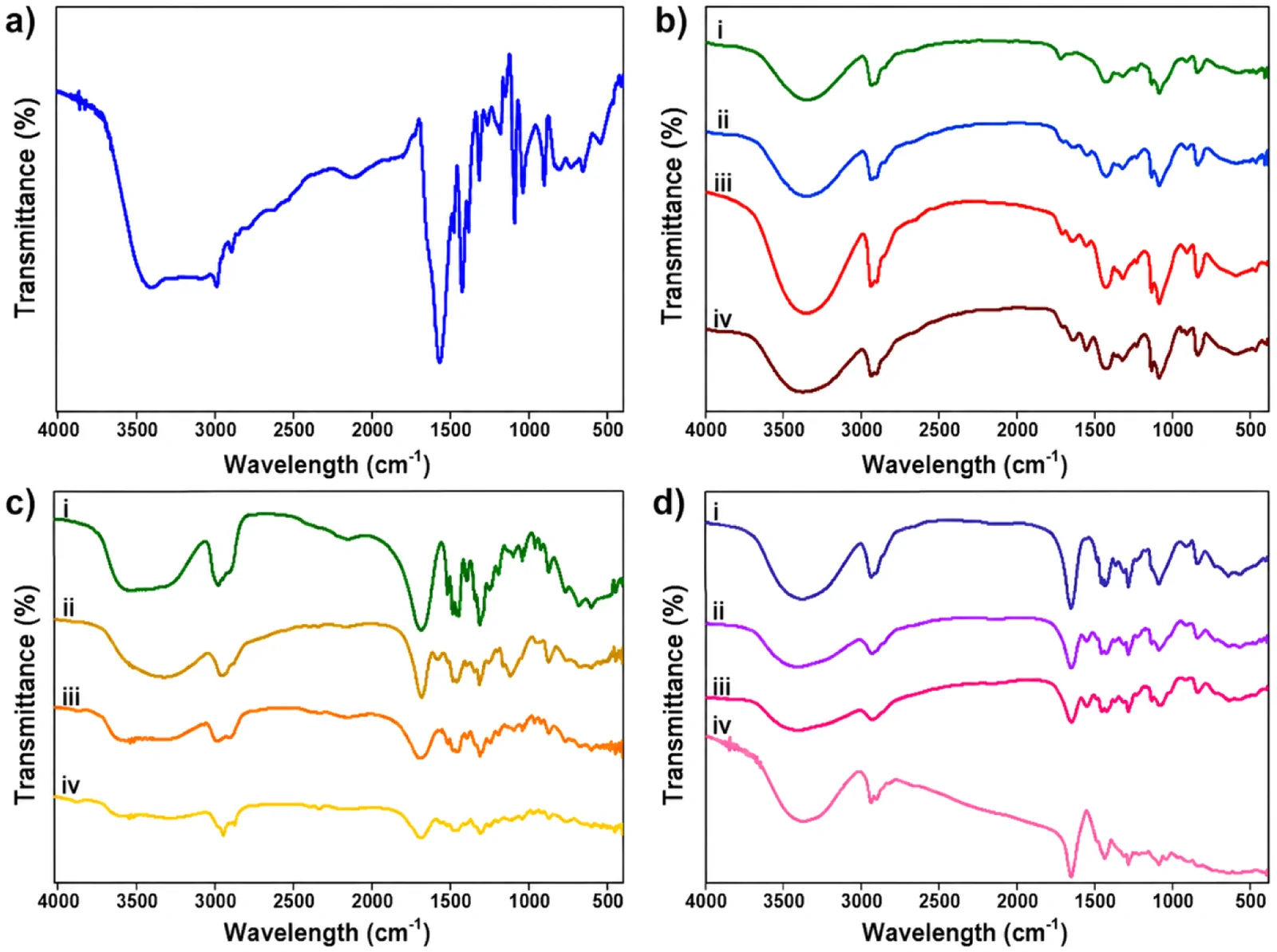
FTIR spectra of (a) the pure 2-HDEAPr PIL and (b–d) fibers: (b) (i) pure PVA, (ii) PVA-0.25 mL PIL, (iii) PVA-0.50 mL PIL, and (iv) PVA-1 mL PIL; (c) (i) pure PVP, (ii) PVP-0.25 mL PIL, (iii) PVP-0.50 mL PIL, and (iv) PVP-1 mL PIL; (d) (i) PVA/PVP blend, (ii) PVA/PVP-0.25 mL PIL, (iii) PVA/PVP-0.50 mL PIL, and (iv) PVA/PVP-1 mL PIL.

The presence of the band at 630 cm^–1^ is attributed to the N–H out-of-plane bending vibration (N–H oop), confirming the incorporation of the protonated amine groups.^63^ The spectral region of 1018–1072 cm^–1^ is characterized by the overlapping of the C–O and C–N vibrational modes.^64^ The band at approximately 1072 cm^–1^ is attributed to the C–O stretching of the primary alcohol groups present in the hydroxyethyl chains of the 2-hydroxydiethylammonium cation. Additionally, in the 1687–1408 cm^–1^ range,^65^ the infrared spectrum exhibits intense absorption bands related to the anion’s identity. The peak at 1408 cm^–1^ is assigned to the symmetric stretching, and 1554 cm^–1^ is assigned to the asymmetric stretching of the COO^–^ groups,^65,66^ providing evidence for the carboxylate functional group in the propionate anion. The broad band centered at 1554 cm^–1^ is still a combined band of the plane N–H bending vibrations.^65,66^ The concomitant presence of these vibrations confirms the acid–base interaction and the successful formation of the 2-HDEAPr PIL.

The protonation of the amine (via proton transfer from propionic acid to diethanolamine) creates a secondary ammonium group. The stretching of this N–H bond does not appear as a sharp peak but rather as a broad and complex band extending across the 3500–2390 cm^–1^ region,^65,66^ including two characteristic bands for NH_2_^+^. The OH stretching vibration is embedded in this band.^65,67^ Particularly, the absorption bands spanning 2754 to 2397 cm^–1^ are technically identified as the ammonium absorption zone, consisting of combination bands and overtones of the NH_2_^+^ group deformations, which result in a characteristic multiplet structure.^68^ Within this high-frequency absorption envelope, sharp and well-defined peaks are identified: at 2883 cm^–1^, corresponding to the C–H symmetric stretching vibrations of the aliphatic chains, and at 2978 cm^–1^, relative to asymmetric stretching for C–H.^65^ The broad absorption band at 3383 cm^–1^ is attributed to the overlap of O–H and N–H stretching vibrations,^65,66^ reflecting the involvement of these groups in a complex network of intermolecular hydrogen bonds with the propionate anion. In this context, the higher frequency region for N–H is characteristic for the stretching modes, while the lower one is characteristic for the bending vibration.^67^

The spectrum of PVA and its complexes (Figure 6b curves i–iv) exhibits typical bands in the regions of 3500–3200 cm^–1^ (O–H stretching),^69^ 3000–2800 cm^–1^ (C–H stretching of alkyl groups),^70^ 1750–1735 cm^–1^ (C=O stretching),^71^ 1461–1417 cm^–1^ (CH_2_ bending), 1150–1085 cm^–1^ (C–O–C stretching),^72^ and 1141 cm^–1^ (associated with ordered (crystalline-like) domains in PVA).^71^ The appearance of peaks around 1560 cm^–1^ in the PVA-0.25 mL, PVA-0.50 mL, and PVA-1 mL samples confirms interactions between the COO^–^ groups of the IL and the CH groups of PVA.^61,73^

A closer inspection of the fingerprint region reveals modifications in the transmission profile located around 1450–1415 cm^–1^ upon increasing the PIL content (from curve i to iv). In pure PVA (curve i), this region is dominated by the bending vibrations of the CH_2_ groups, appearing at 1433 cm^–1^. Interestingly, with the progressive incorporation of 2-HDEAPr PIL, this characteristic band undergoes a shift, migrating, for example, to 1438 cm^–1^ at a PIL content of 0.50 mL. This shift toward higher wavenumbers provides spectroscopic evidence suggesting the successful incorporation of the PIL within the nanofibers and its intimate intermolecular interaction with the PVA. This phenomenon of band displacement and intensity modification is widely reported in the literature as a direct indicator of the intimate interaction of ILs within polymeric systems.^50,53,61,74^

In the spectra of PVP and its complexes with the PIL (Figure 6c, curves i–iv), absorption bands in the range of 1400–1600 cm^–1^ are attributed to the stretching of the COO^–^ bond,^75^ while characteristic bands appear at 1277 cm^–1^ (C–N stretching and CH_2_ bending), 1374 cm^–1^ (CH bending), and 1665 cm^–1^ (C=O stretching).^76^ The transmittance bands between 2750 and 3500 cm^–1^ correspond to CH_2_ and NH_2_^+^ stretching vibrations, with pure PVP exhibiting peaks at 2926 cm^–1^ (symmetric CH_2_ stretching) and 2957 cm^–1^ (asymmetric).^75,76^ The addition of the PIL leads to a broadening of the peak at 1560 cm^–1^ along with a marked reduction in its relative intensity, suggesting an interaction between the C–N group of PVP and the COO^–^ group of the PIL.^61,74^

In the spectra of the PVA–PVP blend (Figure 6d curves i–iv), a peak near 1500 cm^–1^ is also observed in the PVP/PVA-0.25 mL and PVP/PVA-1 mL systems, with lower intensity in PVP/PVA-0.50 mL, reinforcing the interaction between the C–N group (from PVP) and the COO^–^ group (from the PIL).^77^ In curves ii and iii, this peak undergoes a reduction in intensity and a significant broadening. Concurrently, in curve iv, it transitions into a broad, asymmetric band that merges with the neighboring vibrational features. A similar change in the band shape within this region also suggests an interaction between the NH_2_^+^ group of the PIL and the O–H bond of PVA. In the 3100–3600 cm^–1^ region, as the PIL is added (curves ii to iv), this band exhibits a decrease in relative intensity compared to the blend, accompanied by significant broadening and a noticeable shift toward higher wavenumbers.

As indicated by the SEM images (Figure 4), the comparison of FTIR spectra between the pure components and the mixtures confirms the occurrence of chemical interactions between the polymers and the PIL 2-HDEAPr. This is evidenced by subtle shifts in the bands associated with the C=O, O–H, C–N, and NH_2_^+^ functional groups. While the characteristic bands of the PIL (notably the COO^–^ stretching) become more intense with increasing concentration, their integration within the PVA/PVP blend, together with the shifts observed in the polymer carbonyl groups, suggests the formation of a hydrogen-bonding network. These interactions, involving the PIL anion (COO^–^), the cation (NH_2_^+^), and the polar groups of the polymers, promote the reorganization of the polymer chains, resulting in improved phase compatibility and a more homogeneous solution structure, which directly influences the fiber morphology observed in the SEM micrographs.

In the thermal analyses, all the samples showed endothermic peaks (Figure 7) between 62 and 104 °C, and neither of them showed exothermic peaks at the analyzed temperature. This evidence confirms that no oxidation occurred. The endothermic peaks are extremely correlated with the transition glass (*T*_g_) temperature. The *T*_g_ for pure PVP and pure PVA are, respectively, 87.7 and 67.5 °C, in agreement with the literature.^5,78,79^ Particularly for PVA, the decrease in *T*_g_ observed in the pristine material can be attributed to the presence of residual water from the solution, which acts as a plasticizer, leading to an increase in free volume.^80^ The addition of 0.25 and 0.50 mL of PIL led to an increase in *T*_g_. This behavior can be attributed to enhanced intermolecular interactions arising from the presence of side groups in both PVP and PVA, as well as from the ionic liquid structure. The PIL contains polar functional groups (e.g., nitrogen-containing moieties and carbonyl groups), which promote stronger intermolecular interactions and restrict chain mobility, thereby increasing the *T*_g_. On the other hand, for the composition that has 1 mL PIL, there was a different behavior, where the *T*_g_ decreased because the plasticizing effect, taking place, increased the distance between polymeric chains. Thus, the polymer blend formation can be verified using DSC, as indicated by the appearance of a singular endothermic peak, implying the interconnection of both PVP and PVA.^73,78^ *T*_g_ temperature of the blend was recorded at 92.76 °C being a minimal variation, as illustrated in Figure 7c. Therefore, since the observed thermal behavior suggests structural modifications upon PIL incorporation, a reliable quantitative estimation of crystallinity from the present DSC and FTIR data is not feasible due to overlapping transitions and bands, and strong intermolecular interactions.^81^

**Figure 7:**
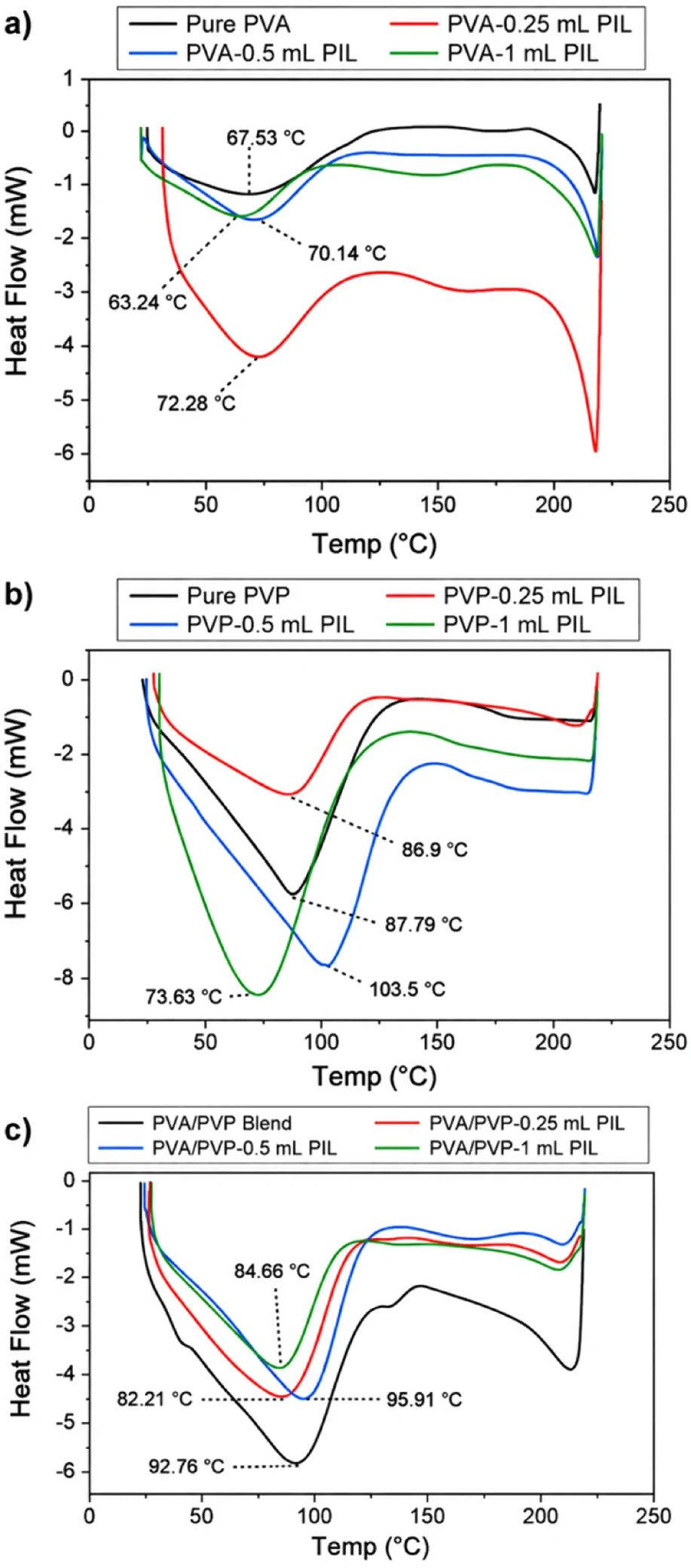
DSC thermograms of the polymer compositions of (a) PVA system, (b) PVP system, and (c) PVA/PVP blend system.

Thus, as can be observed, the minimal addition of 0.25 mL of PIL generated a beneficial effect in reducing the fiber diameter for the pure polymers. The same effect was not observed for the PVA/PVP blend due to more complex interactions between the polymers. Among the investigated conditions, the PVA-0.25 mL PIL sample provided the most optimized membrane morphology, yielding submicrometric fibers (∼0.55 μm) with a narrower diameter distribution. This suggests that the sample can be interesting for applications as scaffolds for biomaterials, e.g., where it has been reported that fibers with smaller diameters produced by SBS are more beneficial for hydroxyapatite growth.^79^ Moreover, increasing the PIL content led to a larger average fiber diameter, indicating that the viscosity effect predominates over the plasticizing effect of the PIL. The higher viscosity limits jet stretching during SBS, resulting in thicker fibers and also in the defects such as casting-like, as shown in Figure 4. In addition, the narrower diameter distribution of the PVA-0.25 mL PIL and PVP-0.25 mL PIL samples may contribute to more homogeneous pore structures of the fibrous membrane, which is beneficial for protein adsorption, cell attachment, and micronutrient release, as demonstrated in the literature.^82–85^ Furthermore, given the use of water-soluble and biocompatible PVA and PVP combined with a low-toxicity PIL, it is reasonable to propose that these membranes may potentially be explored for biomedical scaffolds, drug-delivery systems, and biodegradable coatings, where thinner fibers and structural uniformity are essential for functional performance.

## 4 Conclusions

In this study, aqueous submicrometer fibers of PVP, PVA, and their blends incorporating the protic ionic liquid 2-HDEAPr were successfully synthesized by solution blow spinning. The incorporation of the PIL induced integrated modifications in the chemical, morphological, and thermal properties of the PVA, PVP, and PVA/PVP systems. The minimum amount of 0.25 mL of PIL explored was sufficient to significantly reduce the fiber diameter of the pure polymers due to the balance between the additive plasticizing effect and the solution viscosity. Comparative FTIR analyses, including the spectrum of pure 2-HDEAPr, evidenced intermolecular interactions between the PIL and the functional groups of the polymers, as indicated by the shifts in characteristic vibrational modes compared with those of the pure components. SEM and statistical analyses revealed that concentrations above 0.25 mL of PIL increase the average diameter and promote the formation of casting-type defects. Furthermore, DSC analysis confirmed the plasticizing effect of the 2-HDEAPr PIL, with decreased glass transition temperatures indicating enhanced segmental mobility of the polymer chains. These results collectively demonstrate that the PIL acts as a multifunctional additive in the spinning polymeric solutions, simultaneously tuning the structure, morphology, and thermal stability of the fabricated nano- and submicrofibers, with an ideally low concentration of PIL. Although no direct biological or biodegradation assessment was performed in this study, the present results suggest a promising platform for the development of environmentally benign and potentially biocompatible fibrous materials.

## Supplementary Material



## Notes

Detailed statistical analyses of fiber size and micrographs of polymeric fibers (PDF)
