# Supplementary material for: Nontoxic, Eco-Friendly, Low-Cost Spinning of Submicrometric Fibers from Water-Soluble Polymers Using Protic Ionic Liquids
Source: ACS Omega. 2026 Aug 6;11(32):47302–15. doi: 10.1021/acsomega.5c13582 (PMC13491891; doi:10.1021/acsomega.5c13582)
Supplement: Supplementary file 1 [file ao5c13582_si_001.pdf]

Nontoxic, Eco-Friendly, Low-Cost Spinning of Sub-Micrometric Fibers from Water-Soluble Polymers Using Protic Ionic Liquids

Vinícius D. Silva [a,b], Fabio E. F. Silva [b], Elizeth O. Alves [c], Rebecca S. Andrade [d], Maurício C. Menezes Jr. [d], José E. Soares Filho [b], Miguel Iglesias [e], Daniel A. Macedo [b], Eliton S. Medeiros [b,f], Thiago A. Simões [b,c*]

[a] Department of Fundamental Chemistry, Institute of Chemistry, University of São Paulo, São Paulo/SP, 05508-000, Brazil

[b] Materials Science and Engineering Postgraduate Program, UFPB, 58051-900, João Pessoa, Brazil

[c] Engineering Campus – UACSA, Federal Rural University of Pernambuco (UFRPE), Cabo de Santo Agostinho, PE 54518-430, Brazil

[d] CETENS, Federal University of Recôncavo da Bahia, Feira de Santana, Brazil

[e] Department of Chemical Engineering, Federal University of Bahia, UFBA, 40210-630, Salvador, Brazil

[f] Laboratory of Materials and Biosystems, UFPB, 58051-900, João Pessoa, Brazil

[*]Corresponding author: Prof. Dr. Thiago A. Simões (thiago.araujosimoes@ufrpe.br)

Supplementary Material

a) *Homogeneity of Variance Test*

Brown-Forsythe Test

	DF	Sum of Squares	Mean Square	F Value	Prob>F
Model	3	38.35842	12.78614	134.86497	4.08054E-81
Error	2558	242.51628	0.09481		

At the 0.05 level, the population variances are significantly different.

b) *Homogeneity of Variance Test*

Brown-Forsythe Test

	DF	Sum of Squares	Mean Square	F Value	Prob>F
Model	3	23.17536	7.72512	18.12411	1.28072E-11
Error	2225	948.37157	0.42623		

At the 0.05 level, the population variances are significantly different.

c) *Homogeneity of Variance Test*

Brown-Forsythe Test

	DF	Sum of Squares	Mean Square	F Value	Prob>F
Model	3	19.22627	6.40876	81.7143	3.95043E-50
Error	2208	173.17081	0.07843		

At the 0.05 level, the population variances are significantly different.

Figure S1. Brown-Forsythe test for (a) PVA system, (b) PVP system, and (c) blend PVA/PVP system.

a)

Input Data

	Data	Range
Group Range	[Book2]PVA!A"Group"	[1*:2562*]
Data Range	[Book2]PVA!B"Diameter"	[1*:2562*]

Descriptive Statistics

		N	Min	Q1	Median	Q3	Max
"Diameter"	Pure PVA	641	0.22664	0.70673	0.91506	1.51562	3.88402
	PVA-0.25 mL PIL	765	0.127	0.391	0.516	0.655	1.683
	PVA-0.50 mL PIL	412	0.129	0.423	0.579	0.78425	2.724
	PVA-1.00 mL PIL	744	0.24818	0.6833	0.83421	1.10697	3.09598

Ranks

		N	Mean Rank	Sum Rank
"Diameter"	Pure PVA	641	1670.35179	1070695.5
	PVA-0.25 mL PIL	765	803.88824	614974.5
	PVA-0.50 mL PIL	412	1014.41019	417937
	PVA-1.00 mL PIL	744	1585.47849	1179596

Test Statistics

	Chi-Square	DF	Prob>Chi-Square
"Diameter"	675.38034	3	4.57486E-146

Null Hypothesis:The samples come from the same population.
Alternative Hypothesis:The samples come from different populations.
"Diameter": At the 0.05 level, the populations are significantly different.

Post-hoc Analysis

Dunn's Test

		Mean Rank Diff	Z	Prob	Sig
"Diameter"	Pure PVA PVA-0.25 mL PIL	866.46356	21.87482	2.70784E-105	1
	Pure PVA PVA-0.50 mL PIL	655.9416	14.04286	5.11192E-44	1
	Pure PVA PVA-1.00 mL PIL	84.8733	2.12906	0.19949	0
	PVA-0.25 mL PIL PVA-0.50 mL PIL	-210.52196	-4.6571	1.92417E-5	1
	PVA-0.25 mL PIL PVA-1.00 mL PIL	-781.59026	-20.52007	8.55088E-93	1
	PVA-0.50 mL PIL PVA-1.00 mL PIL	-571.0683	-12.57104	1.82808E-35	1

Sig equals 1 indicates that the difference of the means is significant at the 0,05 level.
Sig equals 0 indicates that the difference of the means is NOT significant at the 0,05 level.

Paired Comparison Plot

Dunn's Test

"Diameter"

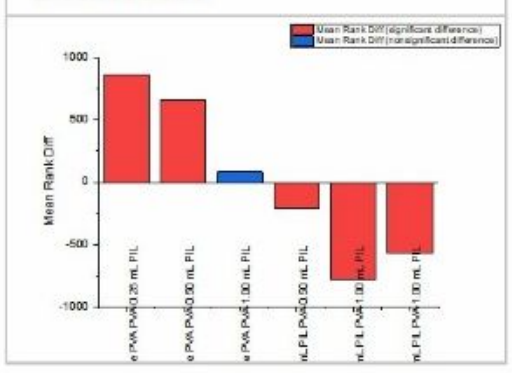

b)

Input Data

	Data	Range
Group Range	[Book2]PVP!A"Group"	[1*:2229*]
Data Range	[Book2]PVP!B"Diameter"	[1*:2229*]

Descriptive Statistics

		N	Min	Q1	Median	Q3	Max
"Diameter"	Pure PVP	719	0.135	0.347	0.508	0.708	12.464
	PVP-0.25 mL PIL	336	0	0.303	0.3785	0.464	0.891
	PVP-0.50 mL PIL	540	0.16577	0.30973	0.52055	0.69133	1.61469
	PVP-1.00 mL PIL	634	0.08	0.353	0.536	0.76125	9.956

Ranks

		N	Mean Rank	Sum Rank
"Diameter"	Pure PVP	719	1193.95758	858455.5
	PVP-0.25 mL PIL	336	774.75298	260317
	PVP-0.50 mL PIL	540	1106.38611	597448.5
	PVP-1.00 mL PIL	634	1213.11356	769114

Test Statistics

	Chi-Square	DF	Prob>Chi-Square
"Diameter"	119.55836	3	9.60618E-26

Null Hypothesis:The samples come from the same population.
Alternative Hypothesis:The samples come from different populations.
"Diameter": At the 0.05 level, the populations are significantly different.

Post-hoc Analysis

Dunn's Test

		Mean Rank Diff	Z	Prob	Sig
"Diameter"	Pure PVP PVP-0.25 mL PIL	419.2046	9.85638	3.8595E-22	1
	Pure PVP PVP-0.50 mL PIL	87.57147	2.38943	0.10125	0
	Pure PVP PVP-1.00 mL PIL	-19.15598	-0.54632	1	0
	PVP-0.25 mL PIL PVP-0.50 mL PIL	-331.63313	-7.41577	7.2554E-13	1
	PVP-0.25 mL PIL PVP-1.00 mL PIL	-438.36059	-10.09354	3.5401E-23	1
	PVP-0.50 mL PIL PVP-1.00 mL PIL	-106.72745	-2.83183	0.02777	1

Sig equals 1 indicates that the difference of the means is significant at the 0,05 level.
Sig equals 0 indicates that the difference of the means is NOT significant at the 0,05 level.

Paired Comparison Plot

Dunn's Test

"Diameter"

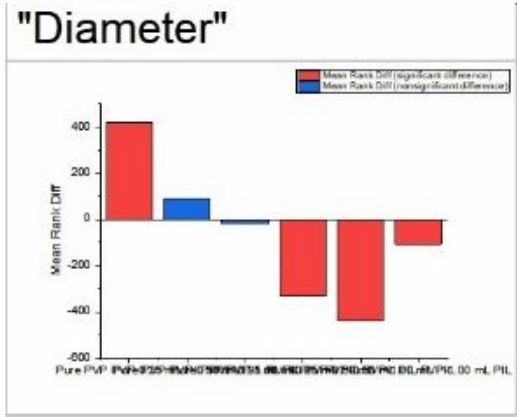

c)

Input Data

	Data	Range
Group Range	[Book2]"PVA-PVP blend"!A"Group"	[1*:2211*]
Data Range	[Book2]"PVA-PVP blend"!B"Diameter	[1*:2211*]

Descriptive Statistics

		N	Min	Q1	Median	Q3	Max
"Diameter"	PVA/PVP blend	771	0.106	0.233	0.325	0.461	1.375
	PVA/PVP-0.25 mL PIL	321	0.057	0.335	0.482	0.653	1.378
	PVA/PVP-0.50 mL PIL	664	0.189	0.407	0.677	1.005	3.739
	PVA/PVP-1.00 mL PIL	455	0.139	0.271	0.396	0.675	2.868

Ranks

		N	Mean Rank	Sum Rank
"Diameter"	PVA/PVP blend	771	776.7166	598848.5
	PVA/PVP-0.25 mL PIL	321	1170.8676	375848.5
	PVA/PVP-0.50 mL PIL	664	1490.62425	989774.5
	PVA/PVP-1.00 mL PIL	455	1056.91099	480894.5

Test Statistics

	Chi-Square	DF	Prob>Chi-Square
"Diameter"	452.14151	3	1.12009E-97

Null Hypothesis:The samples come from the same population.
Alternative Hypothesis:The samples come from different populations.
"Diameter": At the 0.05 level, the populations are significantly different.

Post-hoc Analysis

Dunn's Test

		Mean Rank Diff	Z	Prob	Sig
"Diameter"	?VA/PVP blend PVA/PVP-0.25 mL PIL	-394.151	-9.29471	8.85622E-20	1
	?VA/PVP blend PVA/PVP-0.50 mL PIL	-713.90765	-21.12187	3.00464E-98	1
	?VA/PVP blend PVA/PVP-1.00 mL PIL	-280.19439	-7.42424	6.80571E-13	1
	/P-0.25 mL PIL PVA/PVP-0.50 mL PIL	-319.75665	-7.36789	1.04012E-12	1
	/P-0.25 mL PIL PVA/PVP-1.00 mL PIL	113.95661	2.4489	0.08597	0
	/P-0.50 mL PIL PVA/PVP-1.00 mL PIL	433.71326	11.16304	3.71053E-28	1

Sig equals 1 indicates that the difference of the means is significant at the 0,05 level.
Sig equals 0 indicates that the difference of the means is NOT significant at the 0,05 level.

Paired Comparison Plot

Dunn's Test

"Diameter"

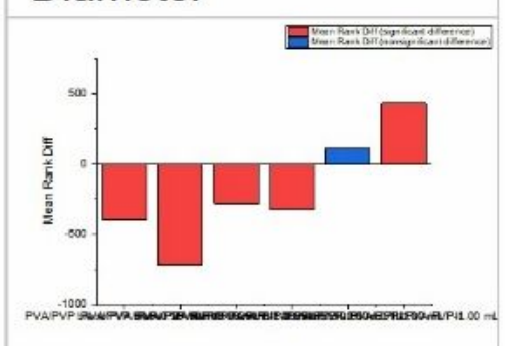

Figure S2. Kruskal-Wallis and Dunn tests for (a) PVA system, (b) PVP system, and (c) blend PVA/PVP system.

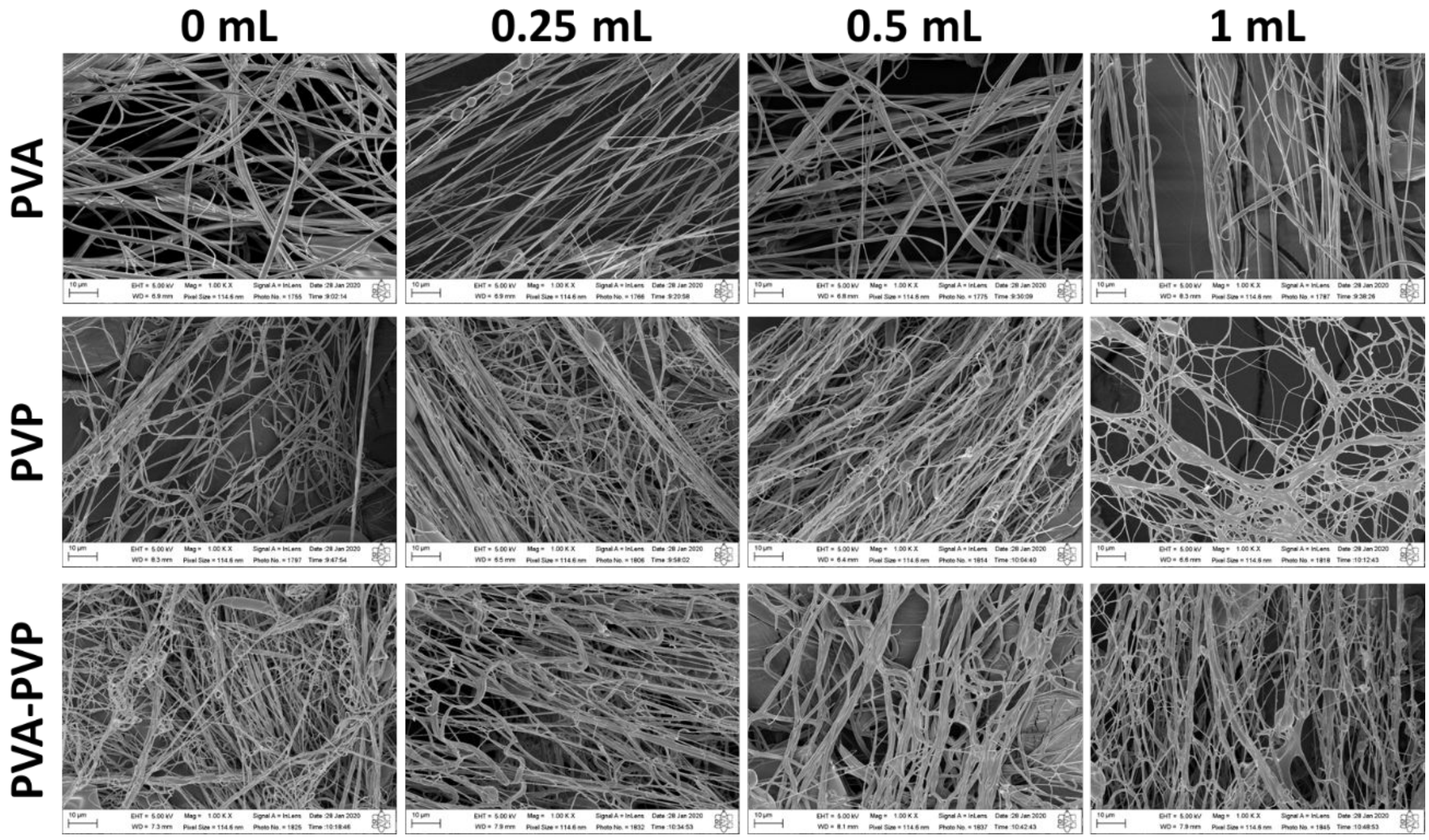

Figure S3. Secondary electron (SE) image at low magnification (1kx).
